# Comparative sedative effects of intravenous etomidate/fentanyl/midazolam versus propofol/fentanyl/midazolam combination for dental treatment of uncooperative children: a randomized clinical trial

**DOI:** 10.1038/s41598-026-59790-3

**Published:** 2026-06-29

**Authors:** Sedighe Mozafar, Masoud Fallahinejad Ghajari, Ahmad Eghbali Zarch, Nastaran Sadat Mahdavi, Aliasghar Soleymani, Morteza Banakar

**Affiliations:** 1 Department of Pediatric Dentistry, Shahed University, Tehran, Iran; 2 Dental Research Center, Research Institute of Dental Sciences, Shahid Beheshti University of Medical Sciences, Tehran, Iran; 3 Pediatric Dentistry Department, Dental School, Shahid Beheshti University of Medical Sciences, Tehran, Iran; 4 Department of Anesthesiology, Medical School, Shahid Beheshti University of Medical Sciences, Tehran, Iran; 5 Department of Anesthesiology, School of Medicine, Mofid Children’s Hospital, Shahid Beheshti University of Medical Sciences, Tehran, Iran; 6 Department of Medical Biotechnology, Shahid Sadoughi University of Medical Sciences, Yazd, Iran; 7 Centre of Molecular Medicine and Diagnostics (COMManD), Saveetha Institute of Medical and Technical Sciences, Saveetha Dental College and Hospitals, Saveetha University, Chennai, India

**Keywords:** Deep sedation, Conscious sedation, Etomidate, Propofol, Fentanyl, Midazolam, Pediatric dentistry, Drug discovery, Health care, Medical research

## Abstract

This study compared the sedative effects of intravenous etomidate/fentanyl/midazolam versus propofol/fentanyl/midazolam combination, with a primary focus on behavioral outcomes assessed by the Houpt scale and hemodynamic safety, for dental treatment of uncooperative children between 3 and 10 years. This patient- and assessor-blinded, crossover clinical trial was conducted on 40 children (27 females, 13 males; mean age, 4.67 ± 1.46 years; mean weight, 15.93 ± 4.2 kg) with negative and very negative behavior, as assessed by the Frankl scale. The children were randomly assigned to 2 groups (*n* = 20). Group 1 received etomidate (0.2 mg/kg), fentanyl (1 µg/kg), and midazolam (0.2 mg/kg) combination in the first treatment session, and propofol (1 mg/kg), fentanyl (1 µg/kg), and midazolam (0.2 mg/kg) in the second treatment session. This order was reversed in group 2. Hemodynamic indices, including the heart rate (HR) and arterial oxygen saturation (SPO2), were monitored during the procedure, and the behavior of children was recorded using the Houpt behavioral rating scale. Data were analyzed using the Mann-Whitney and t tests (α = 0.05). The hemodynamic indices were within the safe range, with no significant difference between the two groups (*P* > 0.05). At the time of injection, a statistically significant but clinically modest difference in SPO2 was observed favoring the etomidate group (99.75% vs. 98.85%, *P* = 0.003); however, mean SPO2 remained above 97% in both groups throughout all procedures. The mean HR showed greater stability with less fluctuation from baseline in the etomidate group compared to propofol (e.g., smaller magnitude of change at injection in the second session, *P* < 0.005 overall for key time points). The two groups were similar regarding the Houpt scale, except during recovery, where the etomidate group had a significantly higher behavior score (7.45 vs. 6.60, *P* = 0.007). Both combinations are highly effective and safe for the intravenous sedation of uncooperative children, providing comparable intra-procedural sedation. However, the etomidate/fentanyl/midazolam combination demonstrated specific advantages regarding intra-procedural heart rate stability and more favorable behavioral scores during the recovery period.

*Trial registration* IRCT20230515058193N1, registeredretrospectively on 22 November 2024 in the Iranian Registry of Clinical Trials (https://www.irct.ir/trial/77589).

## Introduction

Dental fear and the resultant uncooperative behavior are the primary barriers to children receiving high-quality dental care. Dental fear has a close association with the severity and extent of caries and the need for therapeutic interventions^1^. Dental fear and anxiety of children are often maximized at the time of anesthetic injection and dental procedure following their separation from their parents^2^. High levels of fear and anxiety stimulate the sympathetic and parasympathetic systems of children and lead to an increase in their heart rate (HR), blood pressure (BP), and respiratory rate (RR), and inappropriate behaviors^3^. Sedation regimens, such as those targeting gamma-aminobutyric acid (GABA) receptors, can mitigate these responses by depressing the central nervous system, thereby reducing anxiety and stabilizing hemodynamics during procedures^4^.

Evidence shows that both dental clinicians and parents prefer non-pharmaceutical behavioral control methods such as behavior shaping, positive reinforcement, and voice control^5^. Nonetheless, deep sedation or general anesthesia are inevitably required for many anxious, uncooperative children, those with underlying systemic diseases, and disabled or mentally retarded children^6^. It is not always easy to decide between deep sedation and general anesthesia, considering their side effects and complications (although insignificant), the experience and expertise of the medical team, clinical conditions, the severity of caries, the availability of equipment, and the treatment cost^7^. Nonetheless, despite a higher success rate in behavioral control of uncooperative children, general anesthesia is usually considered more invasive and riskier than deep sedation^8^.

Deep sedation enables the provision of an optimal-quality treatment by a dental clinician in a calm environment through partial depression of the central nervous system. Therefore, moderate to deep sedation along with local anesthesia may serve as a low-cost alternative to general anesthesia to control pain and severe anxiety of uncooperative ASA I and II children^9,10^.

An ideal medication and technique for achieving optimal sedation should have a rapid onset of effect, stable effects during the procedure, and a rapid recovery^11^. Evidence shows that intravenous sedation using a combination of several drugs is an effective and safe technique for behavioral control of uncooperative children requiring dental care^12^. To date, several different medications have been used for intravenous sedation. It has been demonstrated that using a combination of different drugs can increase their sedative efficacy and decrease their side effects by minimizing the administered dose of each drug^11^.

Etomidate is a unique gamma-aminobutyric acid type A receptor agonist used for anesthesia induction and sedation, offering hypnotic effects with minimal hemodynamic impact, a fast onset (5–15 s), and a rapid recovery (5–15 min)^13–15^. Its pharmacokinetics make it a suitable drug for continuous infusion^16^. In comparison, propofol, a hypnotic with dose-dependent cardiovascular depression, provides rapid recovery but is associated with greater respiratory risks^17^. Fentanyl is a fast- and short-acting opioid with analgesic effects 60 to 80 times stronger than those of morphine. It has fewer gastrointestinal effects (nausea and vomiting) than other opioids; however, it can cause bradycardia, chest muscle stiffness, and respiratory depression in high doses, which are among its drawbacks^18^. Midazolam is a water-soluble benzodiazepine with hypnotic-sedative effects. It is clinically safer than other hypnotics and causes fewer clinical side effects. However, it causes agitation in some patients, which limits its monotherapy^19^.

Several medications are available for intravenous sedation, each having its own side effects and limitations for use. Thus, it is crucial to find a drug cocktail for this purpose to minimize the dose of individual drugs and their side effects and complications while maximizing the sedation efficacy and safety for dental treatment of uncooperative children. Despite extensive research in this field, an ideal or golden combination for this purpose has yet to be introduced^20,21^. Thus, this study aimed to compare the sedative effects of intravenous etomidate/fentanyl/midazolam versus propofol/fentanyl/midazolam combination for dental treatment of uncooperative children between 3 and 10 years.

## Methods

### Study design

This study was conducted at the Pediatric Dentistry Fellowship Department of Shahid Beheshti University of Medical Sciences between January 2025 and May 2025. The study protocol was approved by the university’s ethics committee (IR.SBMU.DRC.REC.1402.011) and registered in the Iranian Registry of Clinical Trials (registration date: 22 November 2024; registration number: IRCT20230515058193N1).

A randomized crossover clinical trial with patient- and assessor-blinding was designed in which group 1 received etomidate, fentanyl, and midazolam combination in the first treatment session, and propofol, fentanyl, and midazolam in the second treatment session for deep sedation. This order was reversed in group 2. A minimum washout period of 2 weeks was implemented between sessions to minimize carryover effects. The results were reported in accordance with the Consolidated Standards of Reporting Trials.

### Participants, eligibility criteria, and settings

Participants were recruited from 93 children referred to Mofid Children’s Hospital. The inclusion criteria were (I) uncooperative children between 3 and 10 years old with negative and definitely negative behavior according to Frankl’s Behavior Rating Scale^22^, (II) ASA I general health status, and (III) requiring at least two sessions of similar dental treatment under local anesthesia and sedation. Behavior was reassessed using the Frankl scale prior to each session.

The exclusion criteria were (I) underlying systemic diseases, history of asthma or clinically significant allergy, (II) current upper respiratory tract infection (e.g., common cold) or suspected airway obstruction at the time of the dental procedure, and (III) known contraindication to any study medication (e.g., adrenal insufficiency for etomidate, hypersensitivity to propofol or its components, or severe hepatic/renal insufficiency), as determined by the anesthesiologist.

The sample consisted of 40 eligible children presenting to the Pediatric Dentistry Fellowship Department of Shahid Beheshti University of Medical Sciences.

### Interventions

After obtaining written informed consent from the parents, the children underwent dental examination to determine their treatment needs, and a thorough medical examination by an anesthesiologist. The parents received the necessary instructions verbally and in writing upon the admission of their children. Accordingly, the children had to refrain from eating solid foods and milk for 6 h prior to sedation, and drinks for 3 h before sedation. The children’s vital signs, including HR and arterial oxygen saturation (SPO2), were recorded in the operating room. They were also weighed using a digital scale.

With the help of a parent, the children were seated on the dental chair to receive a venous catheter. The children were then randomly assigned to 2 groups (*n* = 20). In the first group, children underwent deep sedation using etomidate (0.2 mg/kg; Aburaihan Pharmaceutical Co., Iran), fentanyl (1 µg/kg; Aburaihan Pharmaceutical Co., Iran), and midazolam (0.2 mg/kg; Aburaihan Pharmaceutical Co., Iran) combination in the first treatment session, which an anesthesiologist administered. During treatment, etomidate administration was continued at an approximate dosage of 150 µg/kg/hour for sedation maintenance, as recommended by the anesthesiologist and pedodontist. In the second treatment session, this group received propofol (1 mg/kg; B. Braun, Germany), fentanyl (1 µg/kg), and midazolam (0.2 mg/kg) for deep sedation. During the course of treatment, supplemental propofol/midazolam was administered as a bolus when clinically indicated by the anesthesiologist and pedodontist based on predefined criteria: purposeful movement, crying, or resistance to treatment that interfered with procedural progress. The supplemental bolus dose consisted of propofol 0.5 mg/kg and midazolam 0.05 mg/kg, administered intravenously over 30–60 s. Supplemental doses were repeated as needed at intervals of no less than 3 min, with a maximum of three supplemental doses permitted per session. The decision to administer a supplemental bolus was made jointly by the anesthesiologist and the pedodontist, who assessed the child’s level of sedation and behavioral response to ongoing treatment. In the etomidate group, sedation was maintained by continuous infusion at an approximate dosage of 150 µg/kg/hour, with no supplemental boluses administered as the continuous infusion provided adequate sedation throughout the procedure. This intermittent bolus approach was chosen due to propofol’s rapid onset and short duration of action, which allows for quick titration of the sedation level for relatively short procedures. In contrast, the continuous infusion of etomidate was chosen to maintain a more constant sedative plane. In the second group (*n* = 20), this order was reversed, and the children underwent deep sedation with propofol, fentanyl, and midazolam with the aforementioned doses in the first treatment session, and etomidate, fentanyl, and midazolam with the aforementioned doses in the second treatment session. The children’s vital signs were monitored throughout the procedure from baseline to discharge, and included HR and SPO2. For this purpose, a calibrated pulse oximeter was placed on the patient’s finger. Also, oxygen was administered with a flow rate of 5 L/minute through a nasal cannula to support respiration. Next, 2% lidocaine with 1:100,000 epinephrine (Darupakhsh, Iran) was injected for local anesthesia. Each procedure took approximately 30 min. The behavioral pattern of children including drowsiness, movement, and crying were recorded at baseline (T0), at the time of intravenous catheter insertion (T1), anesthetic injection (T2), first 15 min after the treatment onset (T3), second 15 min after the treatment onset (T4), and at the time of discharge (T5) using the Houpt behavioral rating scale by an independent pedodontist. The Houpt scale was applied as a composite measure summing scores from multiple behavioral domains: sleep (0–2 points: 0 = eyes open, 1 = eyes intermittently closed, 2 = eyes continuously closed), movement (0–3 points: 0 = violent movement interrupting treatment, 1 = continuous movement making treatment difficult, 2 = moderate movement not interfering with treatment, 3 = no movement), and crying (0–3 points: 0 = hysterical crying, 1 = continuous crying making treatment difficult, 2 = intermittent crying not interfering with treatment, 3 = no crying). The total Houpt score thus ranges from 0 to 8, with higher scores indicating more favorable behavior. Additionally, overall procedural success was rated on a scale from 1 (poor: abandonment of treatment) to 6 (excellent: smooth treatment with no crying or movement)^23^. Furthermore, the pedodontist performing the procedure also observed the patient’s behavior during the procedure and the relevant information was recorded by an assistant. HR and SPO2 were also recorded during the entire process.

After completing treatment, the children were transferred to the recovery room, accompanied by a parent. After observing the discharge criteria including stability of cardiovascular and respiratory function and the ability to respond to stimuli, the children were discharged by the anesthesiologist.

### Outcomes (primary and secondary)

The primary outcome of the study was behavioral success, serving as a clinical proxy for sedation efficacy, as measured by the Houpt behavioral rating scale score. The Houpt scale was chosen as it comprehensively evaluates overall behavior and procedural success, serving as a proxy for sedation efficacy in pediatric dentistry. Secondary outcomes included the safety of the sedation protocols, assessed by monitoring HR and SPO2.

### Sample size calculation

The sample size was calculated to be 20 in each group according to a previous study^24^, assuming α = 0.05, β = 0.2, and study power = 80%, using the following sample size calculation formula:

$$n = \frac{{\left( {Z_{{1 - {\alpha \mathord{\left/ {\vphantom {\alpha 2}} \right. \kern-\nulldelimiterspace} 2}}} + ~Z_{{1 - \beta ~}} } \right)^{2} \sigma ^{2} }}{{\Delta ~\mu ^{2} }}$$

Assuming = ${Z_{{1 - {\alpha \mathord{\left/ {\vphantom {\alpha 2}} \right. \kern-\nulldelimiterspace} 2}}} } =1.96,\:$σ = 0.85, and Δµ = 0.54, via G*Power 3.1 software (Heinrich-Heine University, Düsseldorf, Germany).

### Randomization

The children were randomly assigned to 2 groups by simple randomization. The children were randomly assigned to one of two sequence groups (Group 1 or Group 2) by simple randomization using a computer-generated random number list. The allocation sequence was generated by an independent statistician and concealed in sequentially numbered, opaque, sealed envelopes. The envelope was opened by the anesthesia nurse (who was not involved in the study assessments) only after the child was brought to the operating room and the venous catheter was in place, revealing the drug combination for that session. Allocation of the drug type (etomidate or propofol combination) to the first or second treatment session was thus random and concealed from the enrolling pedodontist.

### Blinding

Due to the distinct physical appearances of propofol (white emulsion) and etomidate (colorless solution) and the different maintenance strategies required (continuous infusion versus intermittent boluses), the attending anesthesiologist could not be blinded to group allocation. However, the pedodontist performing the dental procedure and the assistant recording behavioral data were blinded to the drug administered. To maintain assessor blinding despite these differences, the following measures were implemented: (1) All syringes and infusion lines were covered with opaque tape by an anesthesia nurse who was not involved in data collection or behavioral assessment. (2) The pedodontist and data recorder were positioned behind the patient’s head during drug administration and were not permitted to view the syringes or infusion lines. (3) The anesthesiologist, who was aware of group allocation for safety monitoring, did not participate in behavioral assessments or data recording. (4) The intermittent bolus administration of propofol and continuous infusion of etomidate were both initiated by the anesthesiologist without verbalizing the drug name or administration method in the presence of the pedodontist. These measures ensured that the pedodontist and data recorder remained unaware of the drug administered throughout the procedure. Emergency unblinding was permissible only when clinically necessary.

### Statistical analysis

All statistical analyses were performed using SPSS version 26 (SPSS Inc., IL, USA). The normality of data distribution was analyzed by the Shapiro-Wilk test. To assess potential carryover and period effects in this crossover design, we calculated the period-specific differences for each outcome and compared them between the two sequence groups using independent samples t-tests. Specifically, for each patient, we computed the difference in outcome between the first and second treatment sessions; these differences were then compared between Group 1 (etomidate first) and Group 2 (propofol first) to test for carryover effects. Period effects were evaluated by comparing the mean outcomes of all first-period treatments versus all second-period treatments regardless of drug received. Comparisons were made using paired t-tests and the Wilcoxon signed-rank test for within-subject differences, with Bonferroni correction applied for multiple comparisons across time points (adjusted α = 0.05/k, where k is the number of time points). Data are presented as mean ± standard deviation, and the mean difference (MD) with its 95% confidence interval (CI) is reported for key comparisons. A P-value < 0.05 was considered significant at a 0.05 level of significance.

## Results

### Participant flow

The sample consisted of 40 children, including 27 girls (67.5%) and 13 boys (32.5%), with a mean age of 4.67 ± 1.46 years (range 3–10 years), and a mean weight of 15.93 ± 4.2 kg (range 11–34 kg). Of all children in the first treatment session, 29 (72.5%) were Frankl’s code 1 (very negative behavior), 10 (25% were code 2 (negative behavior), and one patient (2.5%) was code 3, but due to severe nausea, could not undergo conventional treatment and required sedation. In the second treatment session in both groups, 34 (85%) were code 1, 5 (12.5%) were code 2, and 1 (2.5%) was code 3.

**Fig. 1:**
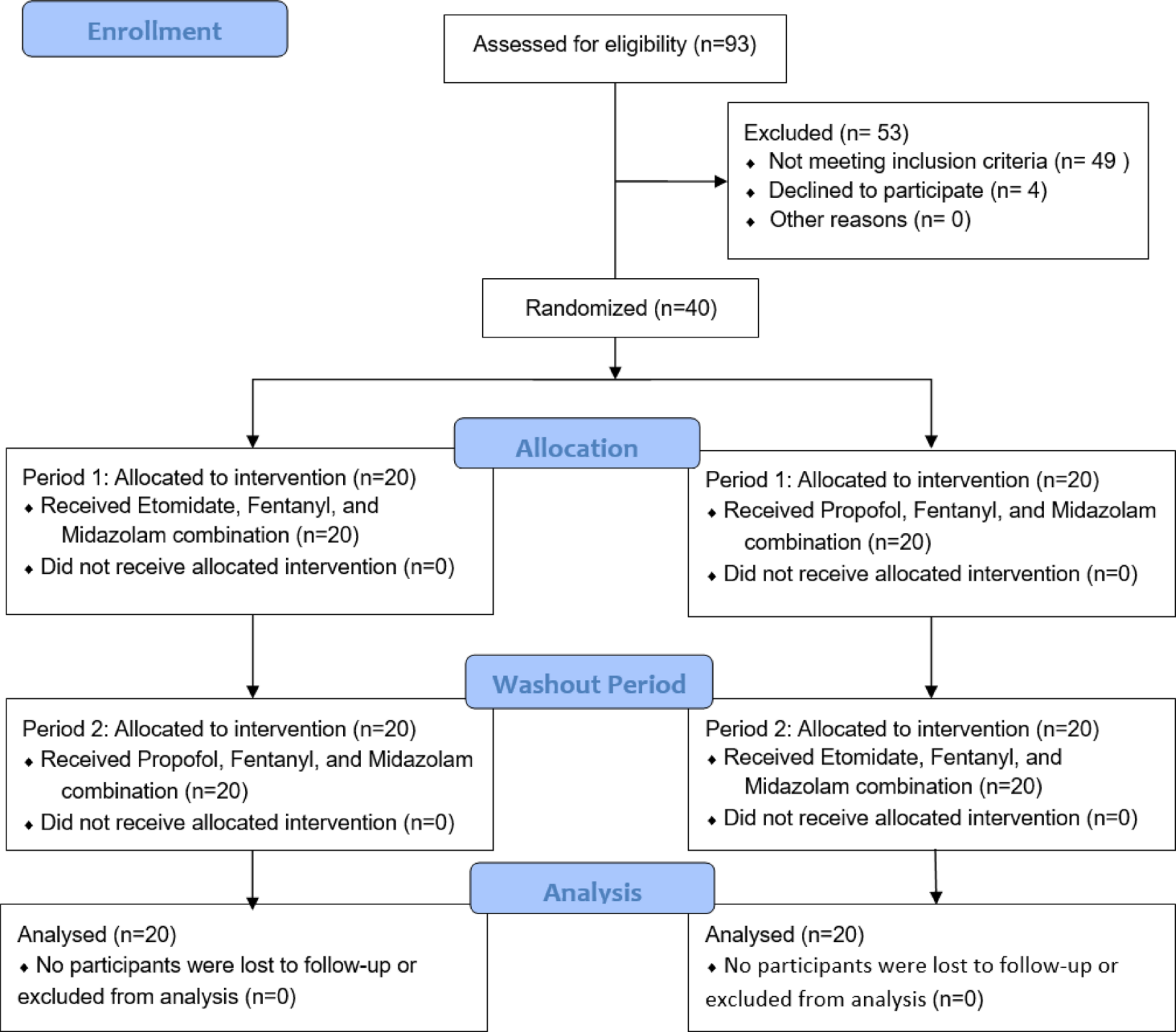
CONSORT flow diagram of patient selection and allocation.

Figure 1 shows the CONSORT flow diagram of patient selection and allocation.

The Shapiro-Wilk test confirmed normal distribution of the carryover and period effect measures in both groups. Independent samples t-test revealed no significant carryover effect, with a mean difference in treatment effects between sequence groups of 0.23 (95% CI -0.48 to 0.94, *P* = 0.508). Similarly, no significant period effect was detected, with a mean difference between first and second period outcomes of 0.19 (95% CI − 0.31 to 0.69, *P* = 0.438). These results confirm that the 2-week washout period was sufficient to eliminate carryover effects, enabling precise comparison between the two treatment regimens.

### Harms

No patients were harmed during the study.

### Subgroup analyses

*SPO2*: Table 1 presents the mean SPO2 at different time points in the two groups. The highest mean SPO2 was noted in the first 15 min after treatment onset in the propofol group, and the lowest mean was recorded at the time of discharge, also in the propofol group, during the first treatment session. In the second treatment session, the highest mean SPO2 was recorded in the first 15 min after the treatment onset in the etomidate group, and the lowest was noted at the time of discharge in the propofol group.

**Table 1:** Mean SPO2 at different time points in the two groups (*n* = 20).

Session	Time	Group	Mean	Std. deviation	Std. error
First session	Baseline	Etomidate	99.40	1.09	0.24
		propofol	97.85	0.87	0.19
	Catheter insertion	Etomidate	99.20	1.39	0.31
		propofol	97.90	0.71	0.16
	Injection	Etomidate	99.00	0.97	0.21
		propofol	99.00	1.37	0.30
	First 15 min	Etomidate	98.75	1.74	0.39
		propofol	99.80	0.61	0.13
	30 min	Etomidate	99.10	1.21	0.27
		propofol	99.40	1.09	0.24
	Recovery	Etomidate	98.25	1.74	0.39
		propofol	97.00	1.25	0.28
Second session	Baseline	Etomidate	98.60	0.94	0.21
		propofol	97.95	1.09	0.24
	Catheter insertion	Etomidate	98.20	1.15	0.25
		propofol	97.85	1.38	0.31
	Injection	Etomidate	99.75	0.55	0.12
		propofol	98.85	1.46	0.32
	First 15 min	Etomidate	99.95	0.22	0.05
		propofol	99.70	0.73	0.16
	30 min	Etomidate	99.65	0.67	0.15
		propofol	99.75	0.78	0.17
	Recovery	Etomidate	97.70	1.17	0.26
		propofol	97.05	1.43	0.32

The Shapiro–Wilk test revealed a normal distribution of SPO2 data at the time of injection and during the first 15 min. Thus, the t-test was used for the comparisons at these two time points. Due to the non-normal distribution of data at other time points, comparisons were made using the Mann–Whitney U test.

**Fig. 2:**
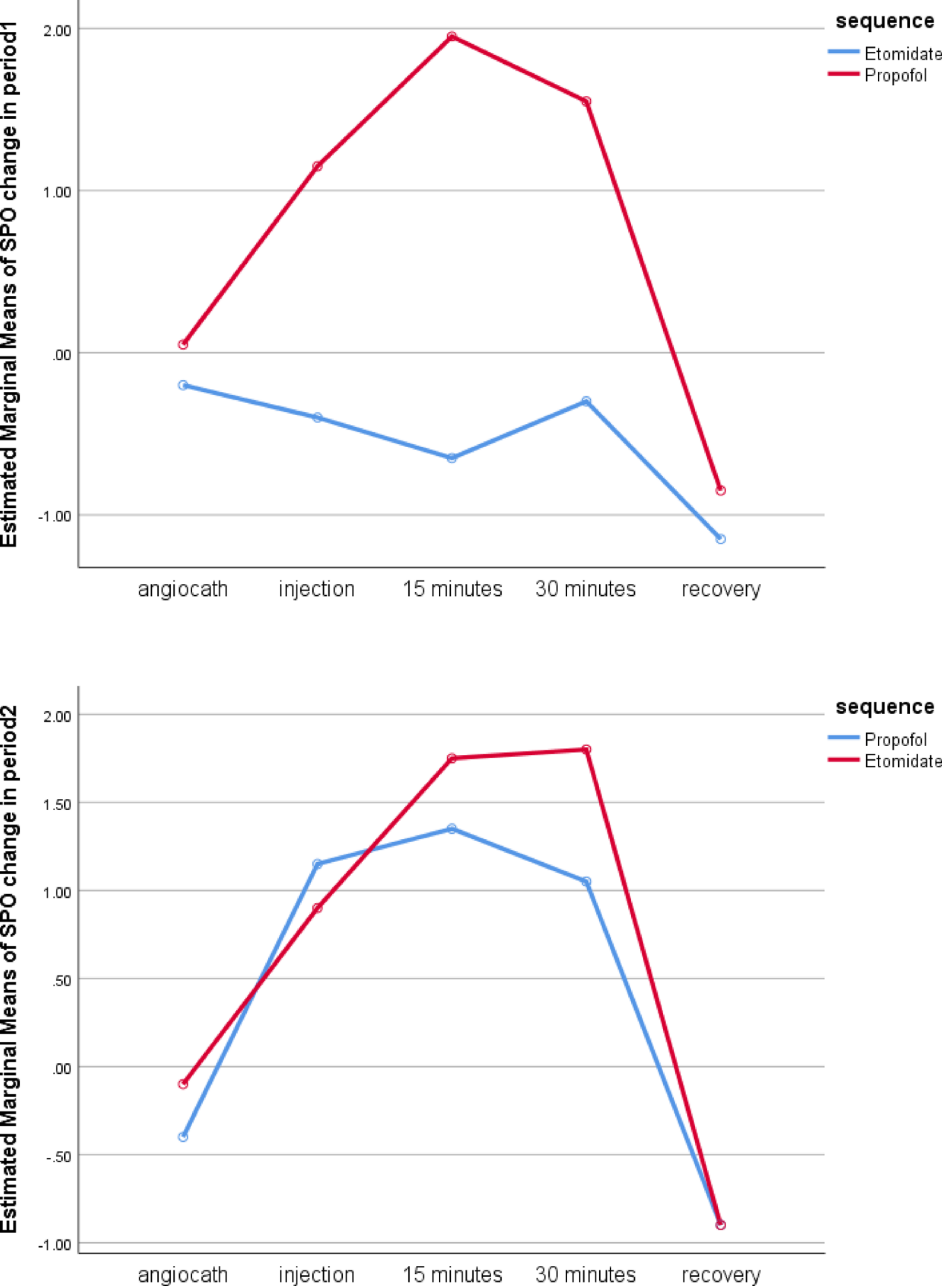
Changes in the mean SPO2 in the two groups in the first (**A**) and second (**B**) treatment sessions.

At the time of injection, the mean absolute SPO2 was significantly higher in the etomidate group compared to the propofol group (99.75% vs. 98.85%; mean difference [MD] 0.90%, 95% CI 0.33–1.47%, *P* = 0.003). It is important to note that while this difference reached statistical significance, the absolute difference was small (0.9%) and both values remained well within the clinically safe range (> 97%). No patient in either group experienced desaturation below 95% at any time point. These values represent aggregated means across both sessions following crossover analysis. Similarly, significant differences favoring the etomidate group were observed at 15 min (*P* = 0.001) and 30 min (*P* = 0.028) after the treatment onset. However, the difference in this regard was not significant between the two groups at the time of catheter insertion (*P* = 0.652) and recovery (*P* = 0.630). Figure 2 illustrates the changes in mean SPO2 for the two groups at various time points during the first and second treatment sessions. As shown, in the first treatment session, SPO2 levels were higher and exhibited greater changes in the propofol group, whereas they showed less change and a more uniform pattern in the etomidate group. In the second treatment session, etomidate provided higher SPO2, and the SPO2 rate in the two groups was close.

*HR*: Table 2 presents the mean HR at different time points in the two groups. As indicated, the highest mean HR was recorded at the time of injection in the etomidate group, and the lowest at baseline in the propofol group in the first treatment session. In the second session, the highest HR was recorded at the time of injection in the etomidate group and the lowest at the time of recovery in the propofol group.

**Table 2:** Mean HR at different time points in the two groups (*n* = 20).

Session	Time	Group	Mean	Std. deviation	Std. error
First session	Baseline	Etomidate	120.65	7.64	1.71
		propofol	113.90	5.80	1.29
	Catheter insertion	Etomidate	137.65	7.78	1.74
		propofol	134.85	6.05	1.35
	Injection	Etomidate	140.35	9.58	2.14
		propofol	137.75	9.03	2.02
	First 15 min	Etomidate	132.15	10.29	2.30
		propofol	131.05	9.08	2.03
	30 min	Etomidate	133.60	9.04	2.02
		propofol	133.65	12.13	2.71
	Recovery	Etomidate	122.65	8.59	1.92
		propofol	117.10	7.49	1.67
Second session	Baseline	Etomidate	122.30	6.19	1.38
		propofol	118.85	6.17	1.38
	Catheter insertion	Etomidate	141.55	9.56	2.13
		propofol	138.80	6.74	1.50
	Injection	Etomidate	147.65	7.90	1.76
		propofol	133.05	9.62	2.15
	First 15 min	Etomidate	140.45	11.51	2.57
		propofol	123.00	10.79	2.41
	30 min	Etomidate	138.05	8.58	1.91
		propofol	126.55	9.41	2.10
	Recovery	Etomidate	125.15	8.78	1.96
		propofol	113.65	7.22	1.61

**Fig. 3:**
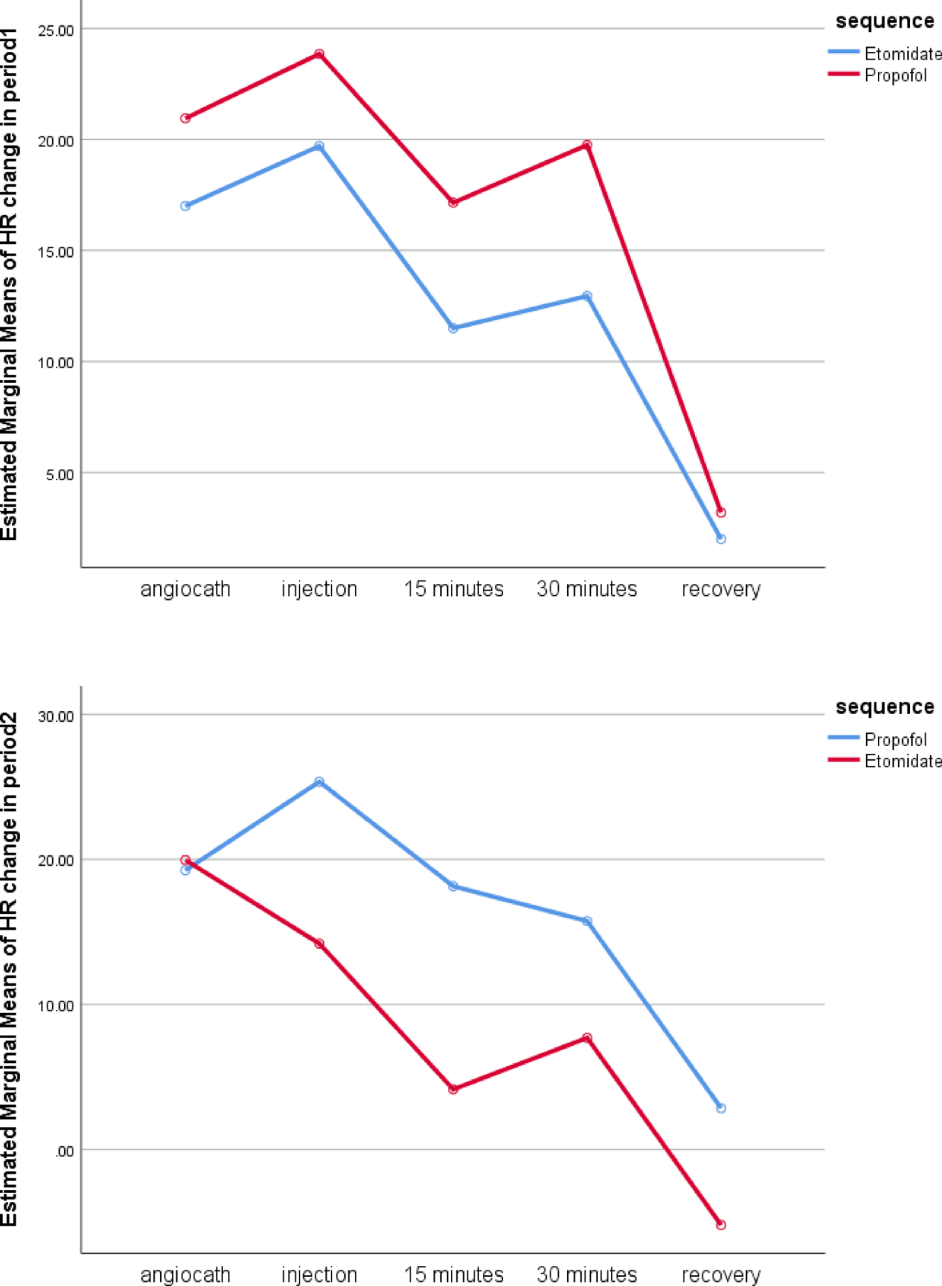
Changes in the mean HR in the two groups at different time points in the first and second treatment sessions.

The two groups were compared by paired t-test, which showed significant differences between the two groups at all time points (*P* < 0.001 for injection time and 15 and 30 min, and *P* = 0.004 for recovery) except catheter insertion (*P* = 0.158). The coefficient of variation for HR was lower in the etomidate group (average 6.5%) compared to propofol (8.2%), indicating greater stability, with all values remaining below 180 bpm. Compared to baseline, the mean HR increased in both the etomidate and propofol groups at the time of injection, after 15 and 30 min, and at the time of recovery. However, the magnitude of HR fluctuation from baseline to peak was less pronounced in the etomidate group, suggesting a more stable heart rate profile throughout the procedure. Figure 3 shows the changes in the mean HR in the two groups at different time points in the first and second treatment sessions. As illustrated, the changes in HR were lower in the etomidate group compared to the propofol group in both treatment sessions.

*Houpt score*: Table 3 presents the mean absolute Houpt scores in the two groups at different time points in the two treatment sessions. The Mann-Whitney test showed no significant difference in overall behavior which included sleep, movement, and crying between the two groups at any time point (*P* > 0.05) except for the recovery period (*P* = 0.007). During the recovery period, the behavioral scores were significantly higher in the etomidate group compared to the propofol group (Mean Difference = 0.85, 95% CI 0.23–1.47, *P* = 0.007), indicating superior behavioral success during emergence.

**Table 3:** Mean Houpt score in the two groups at different time points in the two treatment sessions (*n* = 20).

Session	Time	Group	Mean	Std. deviation	Std. error
First session	Catheter insertion	Etomidate	0.05	0.22	0.05
		propofol	0.00	0.00	0.00
	Injection	Etomidate	8.75	2.02	0.45
		propofol	8.60	1.53	0.34
	First 15 min	Etomidate	8.75	2.02	0.45
		propofol	8.60	1.53	0.34
	30 min	Etomidate	8.75	2.02	0.45
		propofol	8.60	1.53	0.34
	Recovery	Etomidate	6.30	2.05	0.45
		propofol	6.60	1.53	0.34
Second session	Catheter insertion	Etomidate	0.00	0.00	0.00
		propofol	0.00	0.00	0.00
	Injection	Etomidate	9.50	1.43	0.32
		propofol	8.60	1.53	0.34
	First 15 min	Etomidate	9.50	1.43	0.32
		propofol	8.60	1.53	0.34
	30 min	Etomidate	9.50	1.43	0.32
		propofol	8.60	1.53	0.34
	Recovery	Etomidate	7.45	1.35	0.30
		propofol	6.60	1.53	0.34

## Discussion

This study compared the sedative effects of intravenous etomidate/fentanyl/midazolam versus propofol/fentanyl/midazolam combination for dental treatment of uncooperative children between 3 and 10 years. The results showed no significant difference between the two groups in SPO2 at the time of venous catheter insertion and recovery. It is paramount to emphasize that both drug combinations resulted in a 100% treatment success rate and maintained all measured physiological parameters within a safe clinical range. Therefore, both protocols represent viable and effective options for sedating uncooperative children. However, at the time of injection and 15 min after the treatment onset, etomidate significantly increased SPO2 while propofol caused a reduction in SPO2 at these time points. At 30 min, both drug combinations increased SPO2. In general, the two drug combinations had nearly identical effects on SPO2, and this hemodynamic parameter remained above 97% and within the safe range throughout the treatment course in both groups, as SPO2 levels above 95% are considered entirely safe^25^. The statistically significant differences in SPO2 observed at specific time points, while notable from a physiological standpoint, are of limited clinical consequence as mean SPO2 remained above 97% in both groups throughout all procedures and no patient experienced desaturation below 95%. These findings underscore that both sedation protocols provide excellent respiratory safety. In contrast, the more stable HR profile and the significantly superior recovery score in the etomidate group represent findings with clearer clinical relevance, potentially leading to a smoother postoperative period and faster discharge readiness.

Evidence shows that the HR in 2-6-year-olds ranges from 80 to 120 beats/minute; values up to 180 beats/minute are also considered normal following fear, pain, activity, or medication intake and do not usually require therapeutic intervention^26^. The present results regarding HR showed that both drug combinations gradually increased HR over the course of treatment, with all values remaining within accepted pediatric limits (< 180 beats/minute)^26^. The mean HR in the propofol group was higher than that in the etomidate group at most time points, and compared to baseline, HR increased more substantially in the propofol group while remaining relatively stable in the etomidate group. The clinical relevance of these differences warrants careful consideration. While both regimens maintained HR within safe ranges, the greater stability observed with etomidate—evidenced by a lower coefficient of variation (6.5% vs. 8.2%) and smaller fluctuations from baseline—may offer meaningful advantages. In pediatric patients, excessive heart rate variability can reflect sympathetic nervous system activation and increased physiological stress, potentially contributing to a less comfortable procedural experience and influencing recovery quality^27^. Thus, while the absolute HR values were not clinically dangerous in either group, the smoother hemodynamic profile associated with etomidate may translate into a more controlled physiological state during the procedure, which could partially explain the superior recovery behavior observed in this group.

The present results regarding hemodynamic stability of etomidate and minimal cardiovascular effects were in agreement with the results of Hong and Park^27^, Sanri et al.^28^ and Aggarwall et al.^29^ who showed lower respiratory side effects, and more stable and safer hemodynamics in using etomidate compared with propofol. They showed a higher frequency of myoclonus when using etomidate, which did not occur in any patient in the present study.

Evaluation of the general success of sedation according to the Houpt scale and assessment of overall behavior of children revealed that all procedures were successfully performed in all children in both groups with scores 4 (good; treatment was difficult but was accomplished), 5 (very good; with slight crying and movement, treatment was successfully performed), and 6 (excellent; treatment was successfully accomplished with no crying and movement). Thus, the success rate was 100% in both groups with no significant difference except in the recovery period when etomidate resulted in a superior recovery with a higher Houpt score in all patients; this finding was consistent with the results of Kim et al.^15^ who reported superior and fast recovery in the etomidate group as an advantage in short-term treatments. The results regarding the optimal behavior of children in the two groups at other time points were in agreement with the findings of Ahmed et al.^30^ (a 100% success rate in the propofol/fentanyl group). Hong and Park^27^, Sanri et al.^28^ and Aggarwall et al.^29^ reported comparable sedative efficacy of etomidate and propofol for medical treatments^12,28,29^. Our finding that the etomidate group demonstrated a superior behavioral score during recovery is a clinically important advantage for short-term treatments.

The finding that etomidate was associated with significantly better behavioral scores during recovery compared to propofol warrants further consideration, particularly given propofol’s well-documented reputation for rapid, clear-headed recovery and its utility in preventing emergence delirium^31^. Several interrelated factors may explain this observation. First, the administration technique likely played a significant role; the continuous infusion method used for etomidate maintained a more constant sedative plane, avoiding the physiological peaks and troughs associated with the intermittent propofol boluses. Second, etomidate’s unique pharmacokinetic profile—offering a fast onset (5–15 s) and a rapid recovery (5–15 min)—results in predictably swift awakening^32^. Third, the superior cardiovascular stability observed with etomidate, evidenced by significantly reduced heart rate fluctuations and minimal hemodynamic impact, reflects a more controlled sympathetic response throughout the procedure compared to propofol’s dose-dependent cardiovascular depression. This physiological stability could directly translate into calmer behavior upon waking. Finally, minimal impacts on cerebral perfusion and a potentially lower incidence of injection pain with etomidate may reduce negative stimuli, contributing to a smoother emergence profile^33,34^. Future studies incorporating objective measures of recovery quality could provide a more comprehensive understanding of these clinical advantages.

Cardiovascular stability after the injection of a bolus dose is a unique property of etomidate^35^. Thus, etomidate may serve as a suitable alternative to propofol and barbiturates in fast anesthesia induction and sedation. In the present study, the etomidate drug combination was rated more favorable by the operator and parents due to rapid recovery and lower incidence of postoperative pain.

This study had several strengths, including its crossover design, which minimized interindividual variability; standardized drug administration by a single anesthesiologist; and objective behavioral assessment using validated scales (Frankl and Houpt). The inclusion of only ASA I children further enhanced the internal validity of the results.

However, the findings of this study must be interpreted in the context of several limitations. First, while the study employed patient- and assessor-blinding, the attending anesthesiologist could not be blinded due to the distinct physical characteristics of the drugs and the differing administration protocols required. Although the pedodontist and data recorder were blinded, the lack of operator blinding represents a potential source of performance bias that should be acknowledged. Second, and most importantly, the use of different maintenance administration methods (continuous infusion for etomidate versus intermittent boluses for propofol) represents an intervention asymmetry and a major potential limitation. Independent of the distinct pharmacological properties of the drugs, this difference in delivery method may have directly contributed to the observed variations in heart rate stability and recovery behavioral scores. The smoother hemodynamic profile and superior recovery with etomidate could be partly attributable to the steady-state plasma levels achieved with continuous infusion, rather than the drug itself. Furthermore, the exact doses of supplementary propofol boluses administered for maintenance were not systematically compared between the groups, which limits our ability to draw definitive conclusions about total drug requirements. Third, the sample size, while adequately powered for the primary outcome, was relatively small (*n* = 40) and derived from a single center. This limits the generalizability of our findings to other populations and settings. Fourth, our outcome assessment was limited to short-term, intra-procedural, and immediate post-procedural parameters. We did not assess longer-term outcomes such as delayed neurobehavioral effects, parental satisfaction beyond discharge, or the incidence of rare adverse events like adrenal suppression associated with etomidate infusion. Future multicenter studies with larger sample sizes, standardized drug administration protocols (e.g., using target-controlled infusion for both drugs), and longer-term follow-up are recommended to confirm these findings and provide a more comprehensive understanding of the relative advantages of these sedative combinations in pediatric dentistry.

Finally, it should be acknowledged that this trial was registered retrospectively in the Iranian Registry of Clinical Trials on 22 November 2024 (IRCT20230515058193N1), after the completion of the recruitment period (August–October 2024). While the study protocol, ethical approval, and conduct adhered to established standards, the retrospective nature of the registration represents a procedural limitation that should be taken into account when interpreting the findings.

## Conclusion

Both etomidate/fentanyl/midazolam and propofol/fentanyl/midazolam combinations achieved 100% procedural success and maintained hemodynamic parameters within safe ranges, confirming their effectiveness and safety for intravenous sedation of uncooperative children. Etomidate/fentanyl/midazolam was associated with greater heart rate stability and superior behavioral scores during the recovery period, suggesting potential advantages for postoperative recovery quality. However, intra-procedural sedation efficacy was equivalent between the two regimens.

## Data Availability

The datasets generated and/or analyzed during the current study are available from the corresponding author upon reasonable request.
